# Clinical associations of portal-based disease in MASLD: proposal of a new histological scoring system

**DOI:** 10.1007/s00428-025-04087-5

**Published:** 2025-04-10

**Authors:** Daniela S. Allende, Cynthia D. Guy, David E. Kleiner, Danielle Carpenter, Ryan M. Gill, Oscar Cummings, Melissa Contos, Matthew Yeh, Patricia Belt, Laura A. Wilson, Mark Van Natta, Cynthia Behling, the NASH Clinical Research Network

**Affiliations:** 1 Cleveland Clinic, 9500 Euclid Avenue, Cleveland, OH L2544195 USA; 2 Duke University Medical Center, Durham, NC USA; 3 National Cancer Institute, Bethesda, MD USA; 4 Louis University, St. Louis, MO USA; 5 University of California San Francisco, San Francisco, CA USA; 6 Indiana University, Indianapolis, IN USA; 7 Virginia Commonwealth University, Richmond, VA USA; 8 University of Washington, Seattle, WA USA; 9 Johns Hopkins Bloomberg School of Public Health, Baltimore, MD USA; 10 University of California San Diego, San Diego, CA USA

**Keywords:** MASLD, MASH, Portal tract, Inflammation, Fibrosis

## Abstract

Portal inflammation (PI) and ductular reaction (DR) in metabolic dysfunction-associated steatotic liver disease (MASLD) have shown associations with disease severity. We developed a histologic categorization of these features to correlate with known features of MASLD. This study proposes a scoring schema for PI, PP and DR, and relates them to histologic and clinical features in children and adults. This expanded scoring system was developed to identify clinically relevant categories and defined criteria for scoring biopsies. In adults (N:483), more severe PI, PP, and DR were associated with older age (*p* ≤ 0.002), and PP and DR were associated with increased alkaline phosphatase (ALP) (*p* ≤ 0.003), GGT (*p* ≤ 0.001), and total bilirubin (*p* ≤ 0.01). More severe PI, PP, and DR were associated with higher NAFLD activity score (NAS), fibrosis stage, and diagnosis of metabolic dysfunction-associated steatohepatitis (MASH) (*p* ≤ 0.05). In children (N:151), PP and DR were associated with younger age (*p* ≤ 0.0001), and elevated AST, ALT, and ALP (*p* ≤ 0.05). More severe PI, PP, and DR were associated with advanced fibrosis stage, and PP and DR were associated with diagnosis of borderline or definite MASH in children (*p* ≤ 0.05). From multivariable ordinal logistic regression analysis, a higher fibrosis stage was independently associated with more severe PI in both adults and children. Interobserver agreement was substantial for PI, PP and DR. The proposed scoring system demonstrated reproducibility and associations between more severe portal-based disease and advanced liver histology, age, and elevated liver enzymes in adults and children. Evaluation of portal disease could provide insight into therapeutic response and disease progression.

## Introduction

Metabolic dysfunction-associated steatotic liver disease (MASLD) is the most common liver disease in children and adults worldwide and affects one quarter of the population. The disease exists as a clinical and histologic spectrum, with inflammation and hepatocyte injury (metabolic dysfunction-associated steatohepatitis or MASH) and scarring leading to significant liver disease in a subset of patients.

The characteristic liver biopsy features of MASH are steatosis, hepatocyte ballooning, and lobular inflammation typically located in the central, perivenular portion of the lobule (zone 3). However, other histologic features may relate to disease progression and patient outcome.

PI was noted two decades ago as part of the histologic picture of moderate and severe MASH in Brunt et al.’s proposal for grading and staging [1]. Other studies have shown PI associated with higher NAFLD Activity Score (NAS), stage 2 or higher fibrosis [2–4], and increased probability of liver-related events, liver transplantation and mortality [2, 3]. Patients with progressive fibrosis tended to have more PI (inflammatory infiltrate contained within the portal stroma) and PP (inflammatory infiltrate at the interface with spilling over into the adjacent parenchyma) [5].

A prior NASH CRN paper (Brunt 2009) [6] used a simplified chronic PI score to correlate PI with age, female gender, steatosis, fibrosis, and other clinical findings with more inflammation. The study classified PI as 0 (none or rare inflammatory cells, 1 (few mononuclear cells generally in more than one portal tract and 2 (at least one portal tract with moderate to marked inflammation and/or a lymphoid aggregate) but did not address PP/interface inflammation or DR. Correlation of laboratory and other clinical features in MASH with PI has been noted in some studies [7] but not in others [4]. Although prior publications suggest a role for PI in MASLD severity, study of PI and periportal changes have been limited by lack of a well-defined tool for evaluation (qualitative scales).

We sought to propose a scoring system to assess PI, PP, and DR and to correlate these features with clinical and other histologic findings in MASLD. The portal scoring system was analyzed separately from the traditional elements of NAS (steatosis, ballooning, and lobular inflammation) for this study to independently assess its relationship with outcome variables.

## Materials and methods

### Study design

The NIH-sponsored NASH Clinical Research Network (CRN) enrolls children and adults with MASLD from 8 NIH-sponsored sites in the US into a comprehensive database (NCT01030484, NCT01061684, and NCT04454463) which includes clinical and demographic information. Written informed consent was obtained from all study participants (children age 8 and over provided written assent, and written informed consent was obtained from parent or guardian), and the study was approved by the institutional review board at each participating site and the data coordinating center. All patients in the network undergo liver biopsy and the biopsies are scored for numerous histologic parameters by the NASH CRN Pathology Committee during quarterly in-person review at a multiheaded microscope. All NASHCRN pathologists (DSA, CG, DK, DC, RG, OC, MC, MY, and CB) participated in the consensus readings over multiple sessions held by the NASH CRN. A minimum of 3 of the 9 pathologists was required to hold a consensus session and most commonly the sessions included 5–6 pathologists. No prior readings generated from other studies were used. The proposed scoring system was applied to the cases included in the study. Some cases were retrospectively evaluated and some cases were prospectively assessed. Consensus scores for each histologic parameter are determined. The consensus interpretation was achieved by discussion of the different “interpretations” and a majority vote will resolve the discrepancy if needed.

A portal changes scoring system was developed based on authors’ own knowledge on the field, anecdotal experiences, and prior publications [6, 8–12]. We applied the scoring to all consecutive NASHCRN enrolled patients with liver biopsies available between December 2019 and June 2022.

The study evaluated 483 adult and 151 pediatric biopsies from the NASH CRN database, including all stages of fibrosis. All cases had complete clinical and laboratory data. Positive antibody dilutions included ANA ≥ 1:40 and SMA ≥ 1:20. AMA was reported as positive or negative by the enrolling center. Medications taken and diagnosis of metabolic syndrome were reported by the enrolling clinical teams as part of the study. For some parameters, adult and pediatric data was not available for all patients and this may be reflected in different information available in the tables presented. Race and ethnicity data were collected based on self-identification, in concordance with prior publications and following U.S. Office of Management and Budget (OMB) guidelines (available at: https://www.census.gov/about/our-research/race-ethnicity.html. Accessed January 2, 2024).

### Histologic assessment

**Fig. 1:**
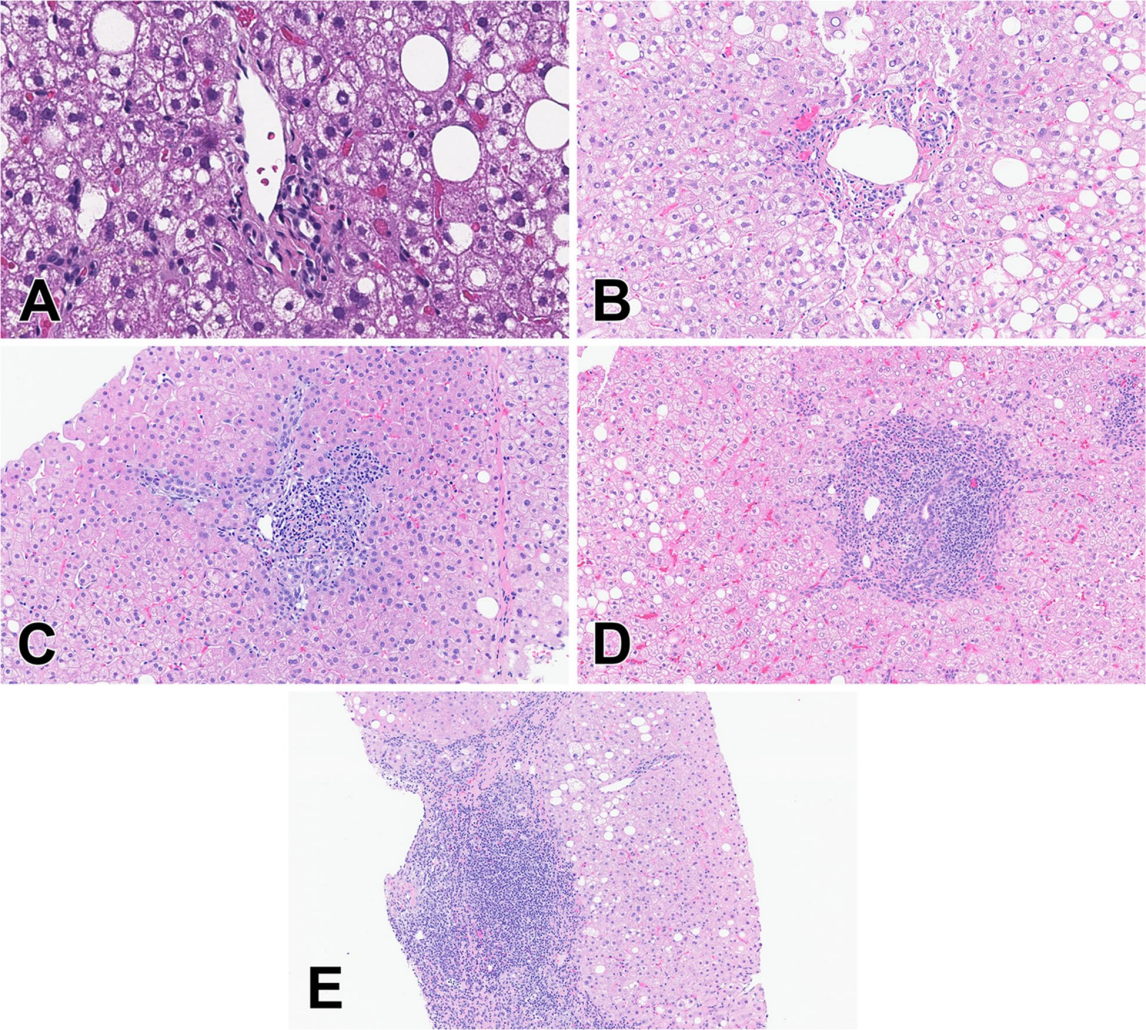
Portal inflammation (PI) score (H&E stains). **A** Score 0: No PI (20 ×), **B** Score 1: minimal PI including sprinkled lymphocytes (10 ×), **C** Score 2: mild PI defined as equal amount of inflammatory cells and matrix (10 ×), **D** Score 3: moderate PI consisting of inflammatory cells that cover the matrix (10 ×), **E** Score 4: severe PI, in which the inflammatory not only covers the matrix but also expands the portal tract (10 ×)

**Fig. 2:**
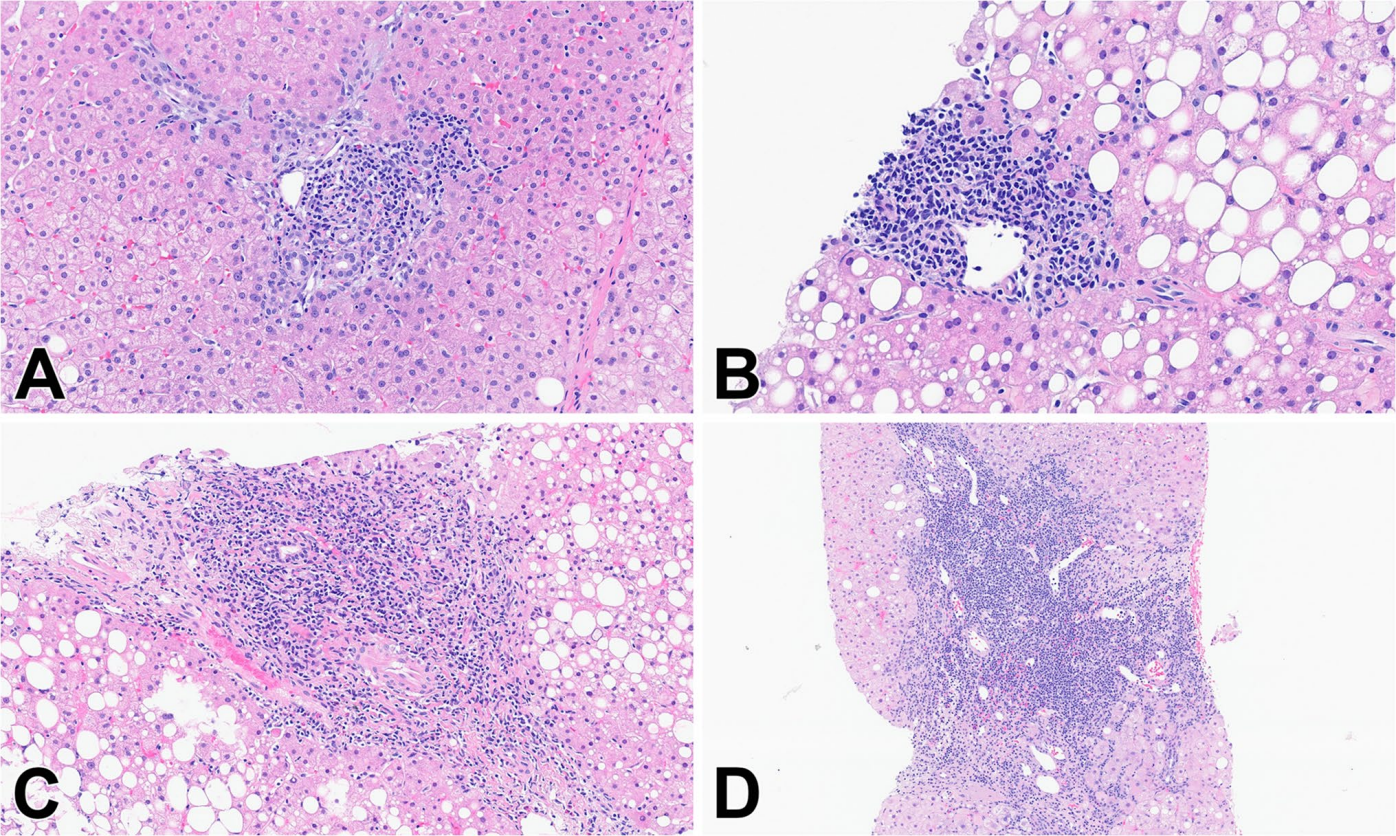
Periportal inflammation (PP) (H&E stains). **A** Score 1: Minimal PP, defined as 1–2 foci of inflammatory cells involving the interface (10x), **B** Score 2: Mild PP characterized by 3–5 foci or less than 1/3 of the portal circumference involved by inflammatory cells (20x), **C** Score 3: Moderate PP with 1/3 to 2/3 of the portal circumference involved by inflammatory cells (10x), **D** Score 4: Severe PP, more than 2/3 of the portal circumference involved (10x)

**Fig. 3:**
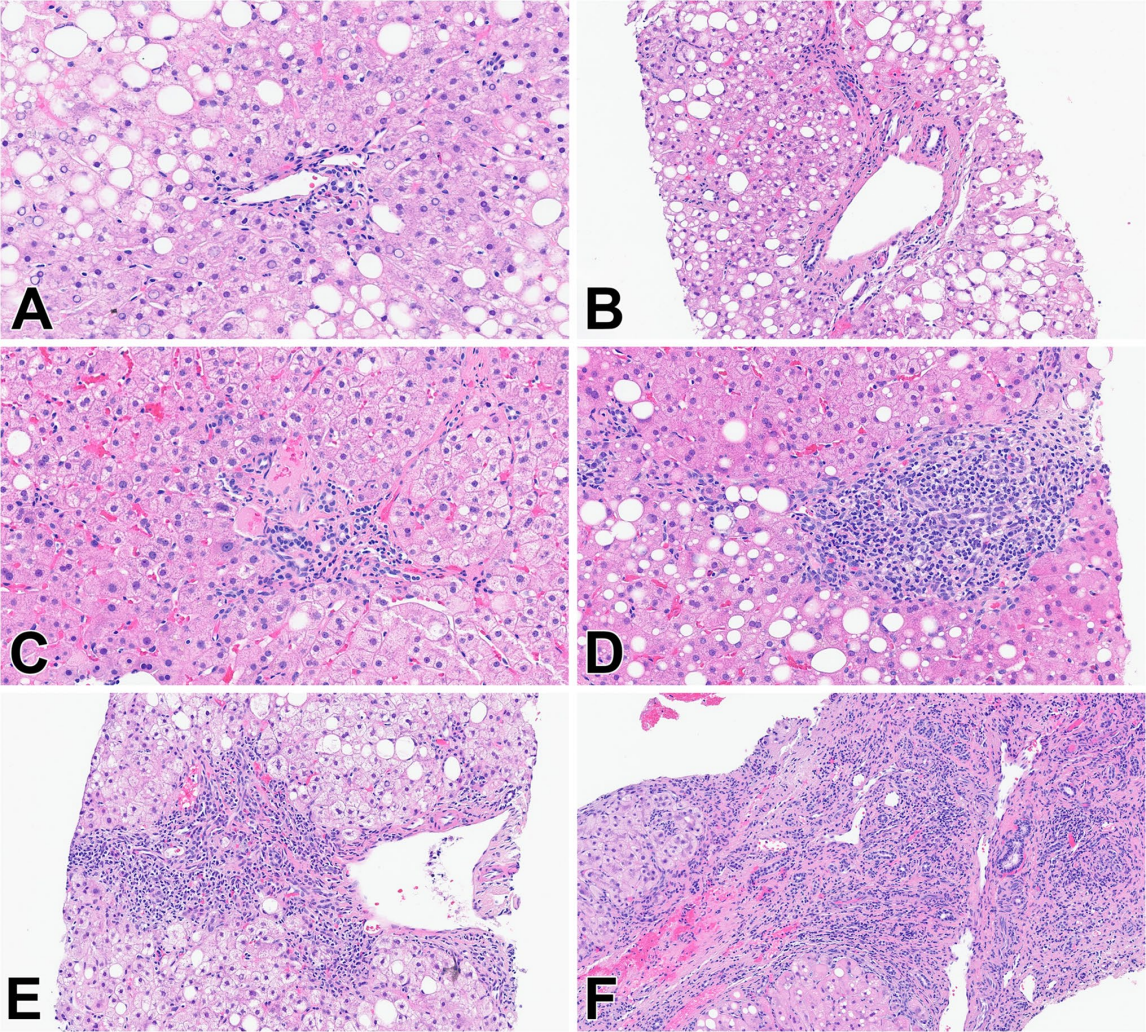
Ductular reaction (DR) (H&E stains). **A** Score 0: no DR (single, native bile duct profile noted) (20 ×), **B** Score 1: minimal DR, in such cases 1–2 bile ductules are seen in portal tracts in addition to the native bile duct (20 ×), **C** Score 2: mild DR, defined as 3–5 bile ductules, in a single layer, mostly at the periphery of the portal tract/interface (20 ×), **D** Score 3: moderate DR including 6–10 bile ductules with multiple bile ductules’ profiles arranged at the periphery (20 ×), **E** Score 4: severe DR demonstrating more than 10 bile ductules with multilayering in early stages of fibrous portal expansion only (10 ×), **F** Score 4: severe DR with multilayering and anastomosing of bile ductules in a case of advanced fibrosis stage (10 ×)

H&E-stained biopsies were scored from 0 to 4 in each of three histologic categories: PI (Fig. 1), PP (Fig. 2), and DR (Fig. 3) (Table 1). Each portal-based histologic feature was scored on the single “most severely affected” portal area for that feature. This was sometimes but not always the same portal tract. The intent behind this approach was to create a more sensitive system, able to capture a more dynamic and broad range of changes and contrasts with some prior studies which averaged portal tract changes and thus blunted the dynamic range.

**Table 1:** Portal inflammation, periportal inflammation, and ductular reaction scoring

**Portal inflammation**	0 None 1 Minimal: sprinkling of inflammation with more matrix visible than inflammation 2 Mild: matrix approximately equal to inflammation 3 Moderate: inflammation covers matrix 4 Severe: inflammation covers matrix and expands the portal tract
Periportal inflammation	0: None 1 Minimal: 1–2 foci of inflammatory cells extending into and at least partially surrounding hepatocytes at the interface 2 Mild: 3–5 foci or less than 1/3 of portal circumference involved by inflammatory cells 3 Moderate: 1/3 to 2/3 of portal circumference involved 4 Severe: more than 2/3 of portal circumference involved
Ductular reaction	0: No ductular reaction, only a single native bile duct profile present 1 Minimal: 1–2 bile ductules in portal tracts 2 Mild: 3–5 bile ductules, arranged in a single layer at the periphery of the portal tract/interface 3 Moderate: 6-10 bile ductules, multiple ductules' profiles arranged as multiple layers around the portal tract periphery 4 Severe: more than 10 bile ductules counted with, multilayers of bile ductules often extending into the fibrous bands or joining portal areas or anastomosing bile ductules

Additional histologic features including steatosis, lobular inflammation, hepatocyte ballooning, diagnosis of MASH, and fibrosis interpreted on trichrome stain were assessed per prior guidelines [12]. Diagnostic categories included no MASLD (patients enrolled based on clinical diagnosis of MASLD, with no histologic evidence of steatosis or steatohepatitis in the biopsy analyzed. This category was not deliberately excluded from our study), MASLD, borderline MASH, and definite MASH. As introduced by this group in a prior publication [12], “borderline” MASH refers to cases that have some but not all features of MASH. Pathologists were blinded to the clinical features of the patients during the biopsy slide review. It is important to clarify that our conclusions are based on correlations identified with the “definite” MASH category and not with the “borderline” MASH cases.

A few practical clarifications regarding the implementation of the scoring system are worth mentioning. First, “DR score 1” refers to 1–2 bile ductules, often at the periphery of the portal tract. These bile ductules are found in addition to the native bile duct, which is most commonly centrally located in the portal tract and accompanying a similarly sized artery. Second, in cases of advanced fibrosis (stages 3 or 4), we evaluated residual portal tracts embedded in the fibrous bands defined as areas within the fibrous bands with an identifiable native bile duct, hepatic artery, and portal vein branch. As a practical point in these cases, assessing the “most severely affected area” of such portal tracts on a 40 × field, centered in a native bile duct allows to define the field to be evaluated.

### Consensus kappa interobserver analysis

A subset of 31 biopsies (21 adult and 10 children) were randomly selected for this purpose. The pathologists were blinded to the selection which included a group representative of our cohort. The cases were rescored as a consensus read by the NASHCRN pathologists to estimate reproducibility of the scoring parameters and kappa statistics. The original scores, not these scores generated in the reproducibility assessment, were used for the study. Discrepancies between the original scores and rescoring were not further analyzed.

### Statistical methods

Descriptive statistics (mean [standard deviation] or number [percent]) are presented for the demographic, clinical, laboratory, and histologic features by PI, PP, and DR categories, and were compared using the Cochran-Armitage test for trend for binary measures and linear regression models for continuous measures. Ordinal logistic regression models with backward selection (*p* < 0.05 for inclusion) for the outcome of expanded portal inflammation score were fit separately for adults and children. Covariates were selected from a candidate set of 13 possible variables: age at biopsy (years), gender, race (white vs. other), Hispanic ethnicity, ALT (U/L), AST (U/L), ALP (U/L), GGT (U/L), steatosis grade (0–3), hepatocyte ballooning (0–2), lobular inflammation grade (0–3), fibrosis stage (0–4), and MASLD diagnosis (not MASLD, MASLD, borderline, or definite MASH). *p*-values < 0.05 were considered statistically significant. Analyses were performed using SAS statistical software, version 9.4 (SAS Institute Inc., Carey, NC) and Stata, release 15.1 (StataCorp).

## Results

**Fig. 4:**
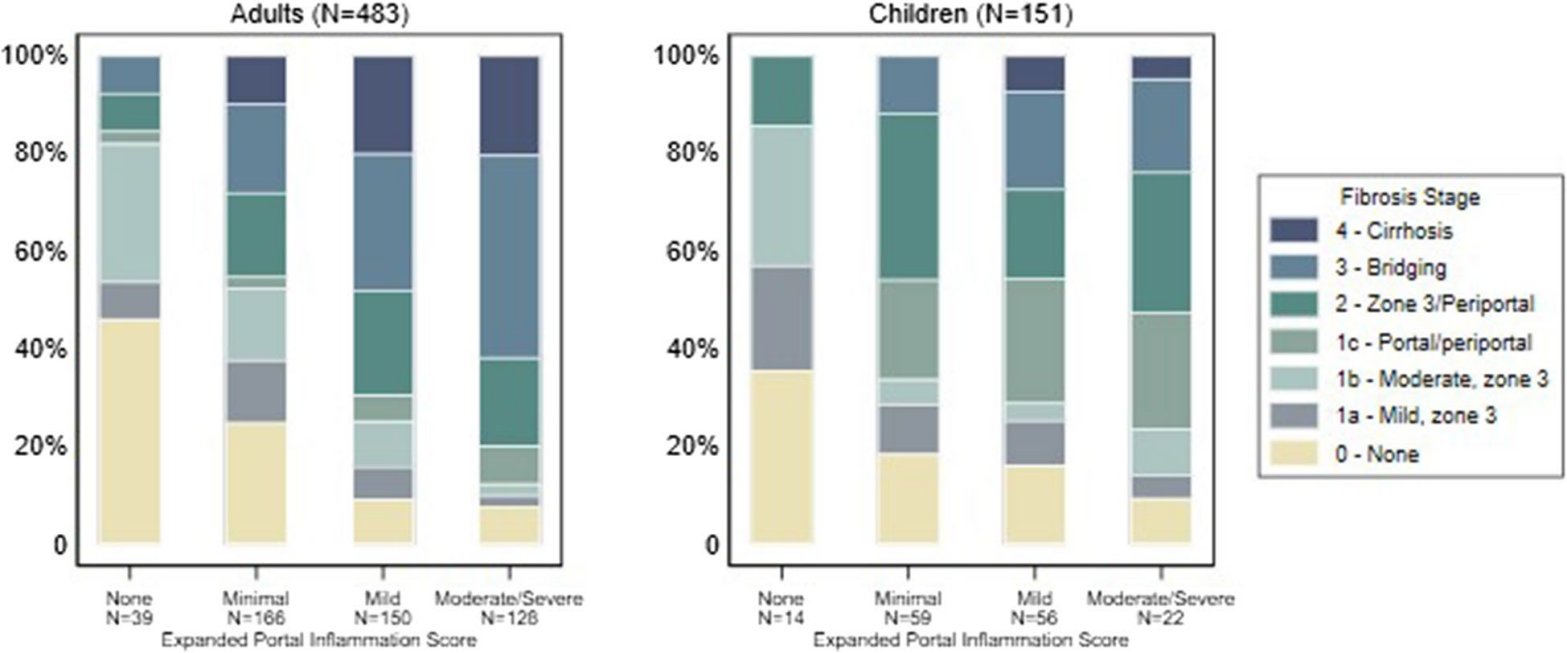
Expanded PI scores (0–4) in relation to fibrosis stage for adults and children

The study included a total of 634 liver biopsies from MASLD patients (adults: 483 and pediatric: 151) (Tables 2, 3, 4, 5, 6, and 7). Biopsies in adults had a mean length of 2.33 cm ± 1 cm (median length 2.1 cm) and in children had a mean length of 2.30 cm ± 0.88 cm (median length 2.2 cm) and were deemed adequate for scoring and staging by the reviewing pathologists. The biopsies represented the full range of fibrosis stage in MASLD and included cases diagnosed as MASH, borderline MASH, and MASLD. A subset of cases in our cohort (No MASLD) corresponded to patients with clinical risk factors for MASLD but no histologic evidence of the disease. The newly developed scoring system for portal changes was applied to cases read between September 2020 and June 2022. Among all biopsies, portal tract changes were common and included moderate to severe PI (150/634, 23%), moderate to severe interface activity (96/634, 15% with more than 1/3 of the portal circumference involved), and moderate to severe ductular reaction (62/634, 9% with 6 or more bile ductules per portal tract) (Fig. 4).

**Table 2:** Selected demographic, laboratory, and histologic features by portal inflammation category in adults (*N* = 483)

	Portal inflammation category				
	None – score 0 (*n* = 39)	Minimal – score 1 (*n* = 166)	Mild – score 2 (*n* = 150)	Moderate to Severe* -Score 3 and 4 (*n* = 128)	*p* value
Demographics					
Age at biopsy, years	47.4 (11.8)	51.2 (12.2)	53.1 (13.0)	54.2 (10.6)	0.01
Metabolic factors					
BMI, kg/m^2^	33.9 (6.5)	34.6 (6.5)	34.8 (6.8)	35.9 (6.5)	0.26
Insulin, μU/mL	28 (37)	24 (23)	33 (30)	30 (20)	0.04
Autoantibody tests†					
ANA, *N* (%) positive	11 (28.2)	47 (28.3)	42 (28.0)	39 (30.5)	0.72
ASMA, *N* (%) positive	8 (20.5)	35 (21.1)	31 (20.7)	30 (23.4)	0.64
AMA, *N* (%) positive	0 (0.0)	4 (2.4)	2 (1.3)	5 (3.9)	0.23
Liver Histology					
NAFLD activity score	3.5 (1.7)	4.1 (1.7)	4.3 (1.8)	4.4 (1.6)	0.02
Steatosis grade	1.6 (0.8)	1.8 (0.8)	1.7 (1.0)	1.6 (0.8)	0.39
Lobular inflammation score	1.3 (0.6)	1.5 (0.7)	1.5 (0.7)	1.6 (0.6)	0.04
Hepatocellular ballooning score	0.6 (0.8)	0.9 (0.8)	1.0 (0.8)	1.2 (0.8)	< 0.0001
Fibrosis stage	0.8 (0.9)	1.6 (1.3)	2.3 (1.3)	2.5 (1.2)	< 0.0001
MASLD diagnosis‡					
No MASLD, *N* (%)	2 (5)	5 (3)	9 (6)	3 (2)	< 0.0001
MASLD, not MASH, *N* (%)	17 (44)	41 (25)	18 (12)	13 (10)	
Borderline MASH, *N* (%)	6 (15)	27 (16)	24 (16)	99 (16)	
Definite MASH, *N* (%)	14 (36)	93 (56)	99 (66)	92 (72)	

^*^3 = moderate inflammation (*n* = 101); 4 = severe inflammation (*n* = 27)

^†^Cochran-Armitage test for trend

^‡^Chi-square

Values are in means (SD) unless otherwise specified

Gender, race, ethnicity, fasting glucose, HOMA-IR, triglycerides, HDL, AST, ALT, alkaline phosphatase, GGT and total bilirubin were not different among the groups (*p* = NS)

**Table 3:** Selected demographic, laboratory, and histologic features by portal inflammation category in children (*N* = 151)

	Portal inflammation category				
	None – score 0 (*n* = 14)	Minimal – score 1 (*n* = 59)	Mild – score 2 (*n* = 56)	Moderate to severe*- scores 3 and 4 (*n* = 22)	*p* value
Demographics					
Age at biopsy, years	14.9 (2.3)	13.5 (2.8)	11.4 (2.5)	13.4 (2.3)	< 0.0001
Metabolic factors					
BMI, kg/m^2^	32.6 (5.4)	33.5 (6.6)	32.4 (6.3)	35.7 (5.3)	0.21
Liver function tests					
Alkaline phosphatase, U/L	171 (128)	202 (123)	273 (113)	209 (86)	0.002
GGT, U/L	66.5 (53.2)	49.2 (30.2)	52.8 (25.0)	47.3 (32.1)	0.27
Autoantibody tests†					
ANA, *N* (%) positive	5 (35.7)	21 (35.6)	16 (28.6)	7 (31.8)	0.55
ASMA, *N* (%) positive	1 (7.1)	7 (11.9)	8 (14.3)	5 (23.8)	0.14
Liver Histology					
NAFLD activity score	4.2 (1.5)	4.5 (1.5)	4.4 (1.4)	4.3 (1.3)	0.93
Steatosis grade	2.2 (0.9)	2.2 (0.9)	2.2 (0.9)	1.9 (0.8)	0.46
Lobular inflammation	1.6 (0.6)	1.6 (0.7)	1.6 (0.6)	1.6 (0.8)	0.90
Portal inflammation	0.1 (0.3)	0.8 (0.4)	1.3 (0.5)	1.6 (0.5)	< 0.0001
Hepatocellular ballooning score	0.4 (0.5)	0.6 (0.8)	0.7 (0.8)	0.8 (0.9)	0.55
Fibrosis stage	0.8 (0.7)	1.4 (0.9)	1.6 (1.2)	1.7 (1.1)	0.03
MASLD diagnosis‡					
No MASLD, *N* (%)	1 (7)	1 (2)	1 (2)	0 (0)	0.70
MASLD, *N* (%)	4 (29)	12 (20)	8 (14)	3 (14)	
Borderline MASH, *N* (%)	4 (29)	23 (39)	27 (48)	8 (36)	
Definite MASH, *N* (%)	5 (36)	23 (39)	20 (36)	11 (50)	

^*^3 = moderate inflammation (*n* = 20); 4 = severe inflammation (*n* = 2)

^†^Cochran-Armitage test for trend

^‡^Chi-square

Values are in means (SD) unless otherwise specified

Gender, race, ethnicity, fasting glucose, Insulin, HOMA-IR, triglycerides, HDL, AST, ALT, and total bilirubin were not different among the groups (*p* = NS)

**Table 4:** Selected demographic, laboratory, and histologic features by periportal inflammation category in adults (*N* = 483)

	Periportal inflammation category				
	None – score 0 (*n* = 160)	1–2 foci – score 1 (*n* = 147)	3–5 foci– score 2 (*n* = 98)	More than 1/3*- scores 3 and 4 (*n* = 78)	*p* value
Demographics					
Age at biopsy, years	50.2 (13.1)	51.1 (11.4)	55.4 (11.4)	54.7 (11.6)	0.001
Race, *N* (%) White†	120 (75)	123 (84)	88 (90)	61 (78)	0.14
Metabolic factors					
BMI, kg/m^2^	33.4 (5.7)	36.5 (7.7)	34.7 (5.6)	35.6 (6.7)	0.0007
Liver function tests					
AST, U/L	44 (33)	46 (30)	44 (21)	56 (33)	0.03
ALT, U/L	66 (57)	62 (44)	55 (33)	63 (36)	0.29
Alkaline phosphatase, U/L	88 (34)	83 (31)	92 (41)	102 (51)	0.007
GGT, U/L	66 (74)	66 (61)	75 (106)	116 (137)	0.0004
Total bilirubin, mg/dL	0.7 (0.5)	0.6 (0.4)	0.7 (0.5)	0.9 (1.1)	0.01
Autoantibody tests†					
ANA, *N* (%) positive	44 (27.5)	44 (29.9)	24 (24.5)	27 (34.6)	0.51
ASMA, *N* (%) positive	36 (22.5)	33 (22.5)	18 (18.4)	17 (21.8)	0.66
AMA, *N* (%) positive	2 (1.3)	2 (1.4)	3 (3.1)	4 (5.2)	0.05
Liver histology					
NAFLD activity score	3.7 (1.8)	4.3 (1.7)	4.4 (1.4)	4.8 (1.6)	< 0.0001
Steatosis grade	1.6 (0.8)	1.8 (0.9)	1.7 (0.9)	1.7 (0.8)	0.12
Lobular inflammation	1.4 (0.7)	1.5 (0.7)	1.5 (0.6)	1.8 (0.7)	0.002
Portal inflammation	0.6 (0.5)	1.2 (0.4)	1.5 (0.5)	1.8 (0.4)	< 0.0001
Hepatocellular ballooning score	0.8 (0.8)	0.9 (0.8)	1.1 (0.8)	1.4 (0.8)	< 0.0001
Fibrosis stage	1.1 (1.1)	2.0 (1.2)	2.5 (1.2)	3.0 (1.1)	< 0.0001
MASLD diagnosis‡					
No MASLD, *N* (%)	10 (6)	4 (3)	3 (3)	2 (3)	< 0.0001
MASLD, *N* (%)	54 (34)	23 (16)	6 (6)	6 (8)	
Borderline MASH, *N* (%)	19 (12)	31 (21)	18 (18)	9 (12)	
Definite MASH, *N* (%)	77 (48)	89 (61)	71 (73)	61 (78)	

^*^3 = 1/3 to 2/3 (*n* = 49); 4 = more than 2/3 (*n* = 29)

^†^Cochran-Armitage test for trend

^‡^Chi-square

Values are in means (SD) unless otherwise specified

Gender, ethnicity, fasting glucose, Insulin, HOMA-IR, triglycerides, and HDL were not different among the groups (*p* = NS)

**Table 5:** Selected demographic, laboratory, and histologic features by periportal inflammation category in children (*N* = 151)

	Periportal inflammation category				
	None – score 0 (*n* = 51)	1–2 foci – score 1 (*n* = 48)	3–5 foci– score 2 (*n* = 34)	More than 1/3*- scores 3 and 4 (*n* = 18)	*p* value
Demographics					
Age at biopsy, years	13.9 (2.4)	13.1 (2.6)	11.8 (2.7)	11.1 (3.0)	< 0.0001
Race, *N* (%) White	27 (53)	36 (75)	25 (74)	14 (78)	0.02
Metabolic factors					
BMI, kg/m^2^	33.8 (6.3)	33.4 (4.5)	32.6 (8.0)	32.8 (6.5)	0.83
Liver function tests					
AST, U/L	64 (41)	97 (62)	90 (72)	149 (134)	0.003
ALT, U/L	112 (86)	167 (107)	161 (137)	243 (216)	0.002
Alkaline phosphatase, U/L	182 (119)	221 (107)	259 (119)	303 (11)	0.0005
GGT, U/L	50.5 (34.6)	50.3 (27.9)	49.8 (30.7)	63.7 (33.4)	0.41
Autoantibody tests†					
ANA, *N* (%) positive	21 (41.2)	12 (25.0)	9 (26.5)	7 (38.9)	0.47
ASMA, *N* (%) positive	6 (11.8)	7 (14.6)	4 (11.8)	4 (23.5)	0.40
Liver histology					
NAFLD activity score	4.1 (1.5)	4.6 (1.6)	4.4 (1.2)	4.6 (1.4)	0.33
Steatosis grade	2.2 (0.9)	2.0 (0.9)	2.1 (0.9)	2.2 (0.8)	0.78
Lobular inflammation	1.5 (0.5)	1.8 (0.8)	1.6 (0.7)	1.6 (0.7)	0.18
Portal inflammation	0.6 (0.6)	1.1 (0.4)	1.3 (0.5)	1.8 (0.4)	< 0.0001
Hepatocellular ballooning score	0.5 (0.7)	0.9 (0.9)	0.6 (0.7)	0.8 (0.9)	0.07
Fibrosis stage	1.0 (0.9)	1.6 (1.0)	1.6 (1.0)	2.1 (1.1)	< 0.0001
MASLD diagnosis‡					
No MASLD, *N* (%)	3 (6)	0 (0)	0 (0)	0 (0%)	0.008
MASLD, *N* (%)	16 (31)	7 (15)	3 (9)	1 (6)	
Borderline MASH, *N* (%)	17 (33)	16 (33)	18 (53)	11 (61)	
Definite MASH, *N* (%)	15 (29)	25 (52)	13 (38)	6 (33)	

^*^3 = 1/3 to 2/3 (*n* = 14); 4 = more than 2/3 (*n* = 4)

^†^Cochran-Armitage test for trend

^‡^Chi-square

Values are in means (SD) unless otherwise specified

Gender, ethnicity, fasting glucose, Insulin, HOMA-IR, triglycerides, HDL, and total bilirubin were not different among the groups (*p* = NS)

**Table 6:** Selected demographic, laboratory, and histologic features by ductular reaction category in adults (*N* = 483)

	Ductular reaction category				
	None – score 0 (*n* = 21)	1–2 bile ductules – score 1 (*n* = 262)	3–5 bile ductules– score 2 (*n* = 148)	6 or more bile ductules*- scores 3 and 4 (*n* = 52)	*p* value
Demographics					
Age at biopsy, years	47.2 (14.2)	50.8 (12.1)	54.3 (12.1)	55.6 (10.3)	0.002
Ethnicity, *N* (%) Hispanic	1 (5)	32 (12)	11 (7)	10 (19)	0.42
Metabolic factors					
BMI, kg/m^2^	34.6 (6.8)	34.3 (6.6)	35.6 (6.6)	35.6 (6.4)	0.06
Liver function tests					
Alkaline phosphatase, U/L	77 (17)	87 (33)	90 (39)	107 (59)	0.003
GGT, U/L	71 (61)	67 (84)	76 (77)	126 (151)	0.0005
Total bilirubin, mg/dL	0.7 (0.4)	0.6 (0.4)	0.7 (0.4)	1.0 (1.3)	0.002
Autoantibody tests†					
ANA, *N* (%) positive	5 (23.8)	73 (27.9)	44 (29.7)	17 (32.7)	0.38
ASMA, *N* (%) positive	4 (19.1)	60 (22.9)	28 (18.9)	12 (23.1)	0.80
AMA, *N* (%) positive	0 (0.0)	4 (1.5)	6 (4.1)	1 (1.9)	0.26
Liver histology					
NAFLD activity score	4.7 (2.0)	4.0 (1.7)	4.3 (1.7)	4.6 (1.6)	0.03
Steatosis grade	2.0 (0.9)	1.7 (0.8)	1.7 (0.9)	1.5 (0.9)	0.12
Lobular inflammation	1.7 (0.8)	1.5 (0.6)	1.6 (0.7)	1.6 (0.7)	0.21
Portal inflammation	0.6 (0.6)	1.0 (0.6)	1.4 (0.5)	1.6 (0.5)	< 0.0001
Hepatocellular ballooning score	1.0 (0.9)	0.8 (0.8)	1.1 (0.8)	1.5 (0.7)	< 0.0001
Fibrosis stage	1.3 (1.0)	1.4 (1.2)	2.5 (1.1)	3.5 (0.6)	< 0.0001
MASLD diagnosis‡					
No MASLD, *N* (%)	2 (10)	9 (3)	5 (3)	3 (6)	< 0.0001
MASLD, *N* (%)	3 (14)	73 (28)	12 (8)	1 (2)	
Borderline MASH, *N* (%)	4 (19)	38 (15)	31 (21)	4 (8)	
Definite MASH, *N* (%)	12 (57)	142 (54)	100 (68)	44 (85)	

^*^3 = 6–10 bile ductules (*n* = 44); 4 = more than 10 bile ductules (*n* = 8)

^†^Cochran-Armitage test for trend

^‡^Chi-square

Values are in means (SD) unless otherwise specified

Gender, race, fasting glucose, insulin, HOMA-IR, triglycerides, HDL, AST, and ALT were not different among the groups (*p* = NS)

**Table 7:** Selected demographic, laboratory, and histologic features by ductular reaction category in children (*N* = 151)

	Ductular reaction category				
	None – score 0 (*n* = 5)	1–2 bile ductules – score 1 (*n* = 94)	3–5 bile ductules– score 2 (*n* = 42)	6 or more bile ductules*- scores 3 and 4 (*n* = 10)	*p* value
Demographics					
Age at biopsy, years	14.8 (2.8)	13.3 (2.7)	12.0 (2.7)	10.6 (1.9)	0.001
Race, *N* (%) White†	1 (20)	54 (57)	38 (90)	9 (90)	< 0.0001
Metabolic factors					
BMI, kg/m^2^	34.2 (7.0)	33.6 (6.1)	32.8 (6.7)	32.6 (6.5)	0.87
HDL, mg/dL	40 (6.2)	37 (8.5)	35 (7.9)	44 (15.2)	0.03
Liver function tests					
AST, U/L	39 (10)	82 (57)	110 (97)	122 (111)	0.04
ALT, U/L	74 (38)	139 (101)	188 (161)	221 (213)	0.04
Alkaline phosphatase, U/L	199 (172)	209 (115)	253 (120)	295 (111)	0.05
GGT, U/L	69.4 (51.2)	48.9 (31.2)	55.7 (29.8	55.5 (31.5)	0.38
Autoantibody tests†					
ANA, *N* (%) positive	2 (40.0)	32 (34.0)	12 (28.6)	3 (30.0)	0.51
ASMA, *N* (%) positive	1 (20.0)	11 (11.8)	8 (19.1)	1 (10.0)	0.72
Liver histology					
NAFLD activity score	2.8 (2.2)	4.5 (1.4)	4.3 (1.4)	4.7 (1.3)	0.08
Steatosis grade	1.4 (1.5)	2.2 (0.8)	2.0 (0.9)	2.3 (0.8)	0.11
Lobular inflammation	1.2 (0.8)	1.6 (0.7)	1.6 (0.7)	1.6 (0.5)	0.59
Portal inflammation	0.2 (0.4)	0.0 (0.5)	1.4 (0.5)	1.6 (0.5)	< 0.0001
Hepatocellular ballooning score	0.2 (0.4)	0.6 (0.8)	0.7 (0.9)	0.8 (0.8)	0.48
Fibrosis stage	1.0 (0.7)	1.2 (0.9)	1.7 (1.0)	2.7 (1.3)	< 0.0001
MASLD diagnosis‡					
No MASLD, *N* (%)	2 (40)	1 (1)	0 (0)	0 (0)	< 0.0001
MASLD, *N* (%)	1 (20)	24 (26)	2 (5)	0 (0)	
Borderline MASH, *N* (%)	1 (20)	33 (35)	23 (55)	5 (50)	
Definite MASH, *N* (%)	1 (20)	36 (38)	17 (40)	5 (50)	

^*^3 = 6–10 bile ductules (*n* = 9); 4 = more than 10 bile ductules (*n* = 1)

^†^Cochran-Armitage test for trend

^‡^Chi-square

Values are in means (SD) unless otherwise specified

Gender, ethnicity, fasting glucose, Insulin, HOMA-IR, triglycerides, and total bilirubin were not different among the groups (*p* = NS)

### Clinical and demographic characteristics in adults

In adults, greater PI, PP, and DR tended to be older patients. There was no significant difference in gender or ethnicity for any of the three portal parameters (Tables 2, 4, and 6). Our population included predominantly white individuals.

PP of any grade correlated with slightly higher BMI (34.7–36.5 versus 33.4, *p* = 0.0007) when compared to those with no PP. The subset of patients with 3–5 foci of PP and those with more than 1/3 of the portal circumference (scores 2, 3, and 4) involved by PP showed higher ALP (92 U/L and 102 U/L vs 83–88 U/L, *p* = 0.007) and GGT (75 U/L and 116 U/L vs 66 U/L, *p* = 0.0004) than others (Table 4). Normal ranges for these values were determined by the submitting laboratories and we cannot comment further on the relative clinical significance (normal versus abnormal). Patients with more than 1/3 of the portal circumference disrupted by inflammation had higher bilirubin levels in comparison to those with less significant PI (0.9 mg/dl vs 0.6–0.7 mg/dL, *p* = 0.01).

Marked DR (grades 3 and 4, 6 or more bile ductules per portal area) correlated with higher bilirubin levels (1 mg/dL versus 0.6–0.7 mg/dL, *p* = 0.002) (Table 6).

ANA, ASMA, and AMA were reported across the spectrum of PI, PP, and DR at levels previously documented in MASLD (*p* = NS). Among the different groups with any PI, mean ANA titer ranged from 289 to 340 IU/mL (dilutions ranging from 1:20 to 1:2560) and mean ASMA titer ranged from 60 to 130 IU/mL (dilutions ranging from 1:20 to 1:640). In patients with any PI, AMA positivity was the least common (2–5% of cases, with a mean titer of 180–320). It was seen more frequently associated with greater PP (*p* = 0.05).

### Clinical and demographic characteristics in children

PI, PP, and DR were worse in younger children. Those with no PI were 14.9 years old while those with any PI were 11.4–13.5 years old (*p* < 0.0001) (Table 3). Greater PP was seen with younger age (PP score 3–4: 11.1 years, PP score 2: 11.8, PP score 1: 13.1 and no PP: 13.9 years, *p* < 0.0001) (Table 5). More pronounced DR was also associated with younger age (DR score 3–4: 10.6 years, DR score 2: 12 years, DR score 1: 13.3 years, and no DR: 14.8 years, *p* = 0.001) and white children (*p* < 0.0001) (Table 7).

Any PI, PP, and DR were associated with increased ALP levels (*p* = 0.002, *p* = 0.0005, *p* = 0.05, respectively) while GGT levels were not different among the groups. Elevated transaminases were identified in children with greater PP and DR (Tables 5 and 7, *p* < 0.05).

### Histologic findings in adults

PI, PP, and DR correlated with higher NAS, higher hepatocyte ballooning score, diagnosis of MASH, and higher fibrosis stage (*p* < 0.05 for all 3 parameters) (Tables 2, 4, and 6).

Moderate to severe PI (scores 3 and 4) was seen in 26% of the adult population in this cohort (128/483). More significant PP (> 1/3 portal circumference involved, scores 3–4) was identified in 16% of cases (78/483). More than 6 bile ductules per portal tract (DR scores 3–4) represented 10% of cases (52/483).

As expected, PP and DR were strongly associated with PI (*p* < 0.0001). Greater PI and PP correlated with greater lobular inflammation (*p* = 0.04 and *p* = 0.002, respectively).

Steatosis did not significantly differ among groups for any of the parameters (PI, PP, or DR).

### Histologic findings in children

Increased PI, PP, and DR were associated with higher fibrosis stage (*p* = 0.03, *p* < 0.0001, and *p* < 0.0001 respectively) (Tables 3, 5, and 7). As seen in adults, PP and DR were strongly associated with PI (*p* < 0.0001).

Any PI was identified in 90% of children (137/151) with 14% of children showing moderate to severe PI (22/151). Significant PP (> 1/3 circumference of the portal tract involved) was seen in 11% of children (18/151). More than 6 bile ductules per portal tract (DR scores 3–4) was identified in 6.6% (10/151).

In addition, more ductular reaction was associated with a definitive diagnosis of MASH (*p* = 0.006). There were no association between PI, PP, and DR and any single histologic feature of MASH (steatosis, hepatocyte ballooning, and lobular inflammation).

### Multivariable ordinal logistic regression (Table 8)

The multivariable ordinal logistic regression analysis for adults revealed that higher fibrosis stage (OR 1.78. 95%CI 1.56–2.03, *p* < 0.001) and Hispanic ethnicity (OR 1.79, 95% CI 1.05–3.07, *p* = 0.03) were independently associated with higher PI scores. Among children, higher fibrosis stage (OR 1.39, 95% CI 1.05–1.85, *p* = 0.02) and younger age at biopsy (OR 0.84, 95% CI 0.76–0.94, *p* = 0.002) were independently associated with higher PI scores.

**Table 8:** Multivariable ordinal logistic regression of expanded portal inflammation by baseline factors

	Multivariable model*		
	Odds ratio	95% CI	***p***
Adult participants (***N*** = 481)			
Fibrosis stage (per one stage increase)	1.78	1.56–2.03	< 0.001
Hispanic vs. non-Hispanic	1.79	1.05–3.07	0.03
Pediatric participants (***N*** = 149)			
Fibrosis stage (per one stage increase)	1.39	1.05–1.85	0.02
Age at biopsy (per one year increase)	0.84	0.76–0.94	0.002

^*^Multivariable model for the outcome of portal inflammation (none, minimal, mild, moderate/severe) was determined from a backward selection model (*p* = 0.05 for inclusion), from a candidate set of 13 covariates, including age at biopsy (years), gender, race (white vs. other), Hispanic ethnicity, ALT (U/L), AST (U/L), alkaline phosphatase (U/L), GGT (U/L), steatosis grade (0–3), hepatocyte ballooning (0–2), lobular inflammation grade (0–3), fibrosis stage (0–4), and MASLD diagnosis (not MASLD, MASLD, borderline MASH, definite MASH). *N* = 481 adults and *N* = 149 children due to missing data

### Reproducibility of the scoring system

Scoring was repeated in 21 adults and 10 children combined. The consensus interobserver agreement for PI was 71% (weighted kappa 0.74), for PP was 52% (weighted kappa 0.66), and for DR was 71% (weighted kappa 0.70).

## Discussion

MASLD is the most common cause of liver disease worldwide, affecting approximately 25% of the population [2].

Portal pathology is a known part of the spectrum of histologic changes seen in MASLD [1–7, 13–15]. However, histologic scoring systems and endpoint evaluation focus on the centrizonal features unique to steatohepatitis.

Currently, scoring systems for portal tract changes in MASLD are rudimentary or borrowed from other liver diseases [1–7]. Prior studies from the NASHCRN have shown portal inflammation in treated MASH biopsies. Mild or more than mild portal inflammation was observed in 82% of cases enrolled in the PIVENS study and 89% of those in the FLINT study in biopsies at baseline [16, 17]. Even though from the anecdotal experience of the authors participating in ongoing clinical trials, we would agree with the fact that treated MASH could show more significant portal inflammation, published data is still conflicting. In the PIVENS study [16], improvement in portal inflammation was associated with improved ALT but no other histologic feature (including fibrosis) (*p* = 0.04). In the FLINT study [17], those with improvement in portal inflammation revealed more fibrosis at baseline (*p* = 0.03). Interestingly, when comparing baseline and end of treatment biopsies, a greater percentage of cases in the group with improvement in fibrosis (versus not responders) revealed portal inflammation in both studies [18]. To highlight another opportunity, one of the most recent clinical trials using Resmetirom has not reported portal inflammation or portal changes after treatment as part of the data included in the manuscript [19]. Other authors have reported persistence and/or increased portal inflammation after gastric bypass surgery as the chosen treatment strategy for MASH [20].

The goal was to develop histologic criteria which were rational, easily learned and used, clinically relevant, and reproducible in children and adults. This portal scoring system is designed to provide a common language and classification scheme for studying these pathology changes in the MASLD research setting. The decision to focus scoring in the “most severely affected” portal tract (i.e., scoring the regions with the highest possible score) in our study rather than averaging the findings was intentional, to increase the sensitivity to identify subtle changes and also for greater reproducibility.

PI was incorporated into the histologic features evaluated and scored for all biopsies in the NASH CRN cases since 2005. The range of portal and periportal findings included in this proposed scoring system captures changes which are more subtle than those observed in other scoring systems used to evaluate portal changes (such as Batts and Ludwig in chronic hepatitis). Portal inflammation in this disease tends to be mild, averaging values (as recommended in other scoring systems such as ISHAK for example), which tends to skew the results towards no significant findings. The proposed system is exploratory of this concept and meant to be sensitive in terms of capturing a wide range of histologic features. PP (interface activity) or DR were not included in the original NAS scoring system but interface activity has been previously observed in MASH [12, 21, 22]. Similarly, DR has been noted in MASH cases, often in the setting of elevated GGT and ALP [7, 21, 23] and even has been identified in arterialized central zones [24].

Despite a formal “ease of use” scale not being applied during the consensus reads, subjectively the criteria were easy to use. A small sample of the cohort was scored twice with pathologists blinded to the initial score or other features of the disease. In this limited group, agreement ranged from 52 to 71% for all three features (PI, PP, and DR). Reasons for discrepancies between scores were not analyzed for this study and the particulars of the scoring schema will require further study and analysis.

In prior studies, portal inflammation was reported [1–7] and “more than mild” portal inflammation [6] correlated with age and features of metabolic syndrome such as BMI, insulin resistance, diabetes, and hypertension in the adult population. In our current study, there were no clear associations with any features of metabolic syndrome, medications, or positive ANA, ASMA, or AMA antibodies as reported in the NASHCRN database. Despite no associations were identified between portal changes and autoantibodies, the analysis of those outlier cases with positive autoantibody expression will be further investigated in an upcoming study. The presence of PI, PP, and DR in younger children is interesting and may reflect the robust inflammatory response often seen in this population. The elevation of transaminases in children with more significant PP and DR poses a question of a potential variant of pediatric MASH that needs to be analyzed in future studies. The current study demonstrated an association between portal changes and fibrosis stage in children and adults. Further, PI in particular was associated with higher fibrosis stage in children and adults also on multivariate analysis. Portal changes were also associated with the diagnosis of MASH in adults but not in children. This is consistent with prior data published by our group [6] and others [3, 15, 21, 22].

Previous studies documented that PI may be associated with disease progression, in particular fibrosis [3, 6, 13–15, 21]. Other authors have shown PI and fibrosis correlate with molecular signatures seen in MASH patients that evolve to fibrosis [14, 25, 26]. Richardson et al. demonstrated an association between PI and DR with fibrosis in the setting of MASLD [23] and hypothesized that activation of the hepatic progenitor cells and cell injury would induce DR leading to fibrosis. More recently, Vilar-Gomez et al. also demonstrated a strong correlation between PI and fibrosis [27]. These authors linked the majority of the effect of *PNPLA3rs738409* on fibrosis to the portal inflammatory pathway.

Our data did not reveal any correlation of portal tract changes with steatosis. Data on this topic may be conflicting. Vilar-Gomez et al. reported a negative association between PI/fibrosis and steatosis, suggesting “burned out steatohepatitis” as a possible interpretation [27]. In contrast, Rakha et al. [7] noted an association of PI with more severe hepatocyte ballooning and steatosis.

We observed mixed results on a possible correlation between lobular inflammation and PI. While lobular inflammation was associated with PI and PP in adults, no association was seen in children with any portal changes. The relative absence of clear correlation between PI and lobular inflammation raises the possibility that there may be independent populations of cells or inflammatory responses. Characterization of the type and characteristics of infiltrating cells is limited [14]. Investigation of the inflammatory cells in both parenchyma and portal areas deserves investigation by advanced imaging, biomarkers or other methods.

Limitations of this study include access to the patients’ clinical and laboratory findings limited to those collected by the NASHCRN, overrepresentation of white population, no detailed medical information regarding diet/ongoing lifestyle modification to treat MASLD, and the fact that the scoring system does not include continuous variables. Given the cross-sectional nature of our cohort, the study lacks long-term follow-up.

Our study reinforces that portal changes are commonly seen in MASLD and associated with a higher likelihood of fibrosis stage in children and adults. Despite ours and others’ publications [28, 29], several questions remain to be further investigated: (1) What may be the mechanisms leading to NASHCRN stage 1c fibrosis in the setting of normal ducts, (2) Is ductular reaction arising from biliary obstruction any different from ductular reaction seen in hepatitic injury such as MASH, (3) DR and PI are associated with progression to fibrosis in MASH and independent of lobular inflammation, but is there a ratio of lobular to portal inflammation that may be relevant? (4) Does persistence of PI after treatment/MASH resolution have clinical significance?

The newly proposed scoring system may help provide a framework in which to study these and other questions about the role of inflammation in MASH and the relative contributions of the most commonly encountered histologic changes in portal tracts in MASLD patients. The link between portal tract pathology (PI, PP, and DR) and fibrosis leaves opens the possibility that one or more of these proposed scales would have independent predictive value in assessing MASH progression or improvement. The scoring system has shown substantial reproducibility among pathologists and correlation with progression of disease, reinforcing its clinical significance. Although further validation may be required, addition of a systematic evaluation of portal changes to MASH scoring could add benefit to existing scoring systems and provide further insight into therapeutic response and clinical progression of disease.

## References

[1] Brunt EM, Janney CG, Di Bisceglie AM, Neuschwander-Tetri BA, Bacon BR (1999) Nonalcoholic steatohepatitis: a proposal for grading and staging the histological lesions. Am J Gastroenterol 94(9):2467–74. 10.1111/j.1572-0241.1999.01377.x 10484010 10.1111/j.1572-0241.1999.01377.x

[2] Younossi ZM, Stepanova M, Rafiq N, Makhlouf H, Younoszai Z, Agrawal R, Goodman Z (2011) Pathologic criteria for nonalcoholic steatohepatitis: interprotocol agreement and ability to predict liver-related mortality. Hepatology 53(6):1874–82. 10.1002/hep.24268 21360720 10.1002/hep.24268

[3] Angulo P, Kleiner DE, Dam-Larsen S, Adams LA, Bjornsson ES, Charatcharoenwitthaya P, Mills PR, Keach JC, Lafferty HD, Stahler A, Haflidadottir S, Bendtsen F (2015) Liver fibrosis, but no other histologic features, is associated with long-term outcomes of patients with nonalcoholic fatty liver disease. Gastroenterology 149(2):389–97.e10. 10.1053/j.gastro.2015.04.043 25935633 10.1053/j.gastro.2015.04.043 PMC4516664

[4] Ballestri S, Nascimbeni F, Romagnoli D, Lonardo A (2016) The independent predictors of non-alcoholic steatohepatitis and its individual histological features: insulin resistance, serum uric acid, metabolic syndrome, alanine aminotransferase and serum total cholesterol are a clue to pathogenesis and candidate targets for treatment. Hepatol Res 46(11):1074–1087. 10.1111/hepr.12656 26785389 10.1111/hepr.12656

[5] Ekstedt M, Franzén LE, Mathiesen UL, Thorelius L, Holmqvist M, Bodemar G, Kechagias S (2006) Long-term follow-up of patients with NAFLD and elevated liver enzymes. Hepatology 44(4):865 17006923 10.1002/hep.21327

[6] Brunt EM, Kleiner DE, Wilson LA, Unalp A, Behling CE, Lavine JE, Neuschwander-Tetri BA, NASH Clinical Research Network- a list of members of the Nonalcoholic Steatohepatitis Clinical Research Network can be found in the Appendix (2009) Portal chronic inflammation in nonalcoholic fatty liver disease (NAFLD): a histologic marker of advanced NAFLD-Clinicopathologic correlations from the nonalcoholic steatohepatitis clinical research network. Hepatology 49(3):809–20. 10.1002/hep.22724 19142989 10.1002/hep.22724 PMC2928479

[7] Rakha EA, Adamson L, Bell E, Neal K, Ryder SD, Kaye PV, Aithal GP (2010) Portal inflammation is associated with advanced histological changes in alcoholic and non-alcoholic fatty liver disease. J Clin Pathol 63(9):790–5. 10.1136/jcp.2010.079145 20819880 10.1136/jcp.2010.079145

[8] Knodell RG, Ishak KG, Black WC, Chen TS, Craig R, Kaplowitz N, Kiernan TW, Wollman J (1981) Formulation and application of a numerical scoring system for assessing histological activity in asymptomatic chronic active hepatitis. Hepatology 1(5):431–5. 10.1002/hep.1840010511 7308988 10.1002/hep.1840010511

[9] Scheuer PJ (1991) Classification of chronic viral hepatitis: a need for reassessment. J Hepatol 13(3):372–4. 10.1016/0168-8278(91)90084-o 1808228 10.1016/0168-8278(91)90084-o

[10] Batts KP, Ludwig J (1995) Chronic hepatitis.An update on terminology and reporting. Am J Surg Pathol 19(12):1409–17. 10.1097/00000478-199512000-00007 7503362 10.1097/00000478-199512000-00007

[11] Ishak K, Baptista A, Bianchi L, Callea F, De Groote J, Gudat F, Denk H, Desmet V, Korb G, MacSween RN et al (1995) Histological grading and staging of chronic hepatitis. J Hepatol 22(6):696–9. 10.1016/0168-8278(95)80226-6 7560864 10.1016/0168-8278(95)80226-6

[12] Kleiner DE, Brunt EM, Van Natta M, Behling C, Contos MJ, Cummings OW, Ferrell LD, Liu YC, Torbenson MS, Unalp-Arida A, Yeh M, McCullough AJ, Sanyal AJ, Nonalcoholic Steatohepatitis Clinical Research Network (2005) Design and validation of a histological scoring system for nonalcoholic fatty liver disease. Hepatology 41(6):1313–21. 10.1002/hep.20701 15915461 10.1002/hep.20701

[13] Mann JP, De Vito R, Mosca A, Alisi A, Armstrong MJ, Raponi M, Baumann U, Nobili V (2016) Portal inflammation is independently associated with fibrosis and metabolic syndrome in pediatric nonalcoholic fatty liver disease. Hepatology 63(3):745–53. 10.1002/hep.28374 26638195 10.1002/hep.28374

[14] Gadd VL, Skoien R, Powell EE, Fagan KJ, Winterford C, Horsfall L, Irvine K, Clouston AD (2014) The portal inflammatory infiltrate and ductular reaction in human nonalcoholic fatty liver disease. Hepatology 59(4):1393–405. 10.1002/hep.26937 24254368 10.1002/hep.26937

[15] Alkhouri N, Mansoor S, Giammaria P, Liccardo D, Lopez R, Nobili V (2014) The development of the pediatric NAFLD fibrosis score (PNFS) to predict the presence of advanced fibrosis in children with nonalcoholic fatty liver disease. PLoS One 9(8):e104558. 10.1371/journal.pone.0104558 25121514 10.1371/journal.pone.0104558 PMC4133235

[16] Sanyal AJ, Chalasani N, Kowdley KV, McCullough A, Diehl AM, Bass NM, Neuschwander-Tetri BA, Lavine JE, Tonascia J, Unalp A, Van Natta M, Clark J, Brunt EM, Kleiner DE, Hoofnagle JH, Robuck PR (2010) Pioglitazone, vitamin E, or placebo for nonalcoholic steatohepatitis. N Engl J Med 362(18):1675–85. 10.1056/NEJMoa0907929 20427778 10.1056/NEJMoa0907929 PMC2928471

[17] Neuschwander-Tetri BA, Loomba R, Sanyal AJ, Lavine JE, Van Natta ML, Abdelmalek MF, Chalasani N, Dasarathy S, Diehl AM, Hameed B, Kowdley KV, McCullough A, Terrault N, Clark JM, Tonascia J, Brunt EM, Kleiner DE, Doo E, NASH Clinical Research Network (2015) Farnesoid X nuclear receptor ligand obeticholic acid for non-cirrhotic, non-alcoholic steatohepatitis (FLINT): a multicentre, randomised, placebo-controlled trial. Lancet 385(9972):956–65. 10.1016/S0140-6736(14)61933-4 25468160 10.1016/S0140-6736(14)61933-4 PMC4447192

[18] Brunt EM, Kleiner DE, Wilson LA, Sanyal AJ, Neuschwander-Tetri BA, Nonalcoholic Steatohepatitis Clinical Research Network (2019) Improvements in histologic features and diagnosis associated with improvement in fibrosis in nonalcoholic steatohepatitis: results from the Nonalcoholic Steatohepatitis Clinical Research Network Treatment Trials. Hepatology 70(2):522–531. 10.1002/hep.30418 30549292 10.1002/hep.30418 PMC6570584

[19] Harrison SA, Bedossa P, Guy CD, Schattenberg JM, Loomba R, Taub R, Labriola D, Moussa SE, Neff GW, Rinella ME, Anstee QM, Abdelmalek MF, Younossi Z, Baum SJ, Francque S, Charlton MR, Newsome PN, Lanthier N, Schiefke I, Mangia A, Pericàs JM, Patil R, Sanyal AJ, Noureddin M, Bansal MB, Alkhouri N, Castera L, Rudraraju M, Ratziu V, MAESTRO-NASH Investigators (2024) A phase 3, randomized, controlled trial of Resmetirom in NASH with liver fibrosis. N Engl J Med 390(6):497–509. 10.1056/NEJMoa2309000 38324483 10.1056/NEJMoa2309000

[20] Barker KB, Palekar NA, Bowers SP, Goldberg JE, Pulcini JP, Harrison SA (2006) Non-alcoholic steatohepatitis: effect of Roux-en-Y gastric bypass surgery. Am J Gastroenterol 101(2):368–73. 10.1111/j.1572-0241.2006.00419.x 16454845 10.1111/j.1572-0241.2006.00419.x

[21] Vespasiani-Gentilucci U, Carotti S, Perrone G, Mazzarelli C, Galati G, Onetti-Muda A, Picardi A, Morini S (2015) Hepatic toll-like receptor 4 expression is associated with portal inflammation and fibrosis in patients with NAFLD. Liver Int 35(2):569–81. 10.1111/liv.12531 24649857 10.1111/liv.12531

[22] Pais R, Charlotte F, Fedchuk L, Bedossa P, Lebray P, Poynard T, Ratziu V, LIDO Study Group (2013) A systematic review of follow-up biopsies reveals disease progression in patients with non-alcoholic fatty liver. J Hepatol 59(3):550–6. 10.1016/j.jhep.2013.04.027 23665288 10.1016/j.jhep.2013.04.027

[23] Richardson MM, Jonsson JR, Powell EE, Brunt EM, Neuschwander-Tetri BA, Bhathal PS, Dixon JB, Weltman MD, Tilg H, Moschen AR, Purdie DM, Demetris AJ, Clouston AD (2007) Progressive fibrosis in nonalcoholic steatohepatitis: association with altered regeneration and a ductular reaction. Gastroenterology 133(1):80–90. 10.1053/j.gastro.2007.05.012 17631134 10.1053/j.gastro.2007.05.012

[24] Gill RM, Belt P, Wilson L, Bass NM, Ferrell LD (2011) Centrizonal arteries and microvessels in nonalcoholic steatohepatitis. Am J Surg Pathol 35(9):1400–4. 10.1097/PAS.0b013e3182254283 21836480 10.1097/PAS.0b013e3182254283 PMC3156381

[25] Swiderska-Syn M, Suzuki A, Guy CD, Schwimmer JB, Abdelmalek MF, Lavine JE, Diehl AM (2013) Hedgehog pathway and pediatric nonalcoholic fatty liver disease. Hepatology 57(5):1814–25. 10.1002/hep.26230 23300059 10.1002/hep.26230 PMC3637920

[26] Hübscher SG (2006) Histological assessment of non-alcoholic fatty liver disease. Histopathology 49(5):450–65. 10.1111/j.1365-2559.2006.02416.x 17064291 10.1111/j.1365-2559.2006.02416.x

[27] Vilar-Gomez E, Pirola CJ, Sookoian S, Wilson LA, Belt P, Liang T, Liu W, Chalasani N (2021) Impact of the association between PNPLA3 genetic variation and dietary intake on the risk of significant fibrosis in patients with NAFLD. Am J Gastroenterol 116(5):994–1006. 10.14309/ajg.0000000000001072 33306506 10.14309/ajg.0000000000001072 PMC8087619

[28] Desmet VJ (2011) Ductal plates in hepatic ductular reactions. Hypothesis and implications. I. Types of ductular reaction reconsidered. Virchows Arch 458(3):251–9. 10.1007/s00428-011-1048-3 10.1007/s00428-011-1048-3 21287200

[29] Desmet VJ (2011) Ductal plates in hepatic ductular reactions. Hypothesis and implications. III. Implications for liver pathology. Virchows Arch 458(3):271–9. 10.1007/s00428-011-1050-9 10.1007/s00428-011-1050-9 21301864

